# Prevalence of Folate Deficiency Amongst Patients in a Secondary Care Outpatient Mental Health Service

**DOI:** 10.1192/j.eurpsy.2026.10590

**Published:** 2026-07-31

**Authors:** C. Pringle, N. Shaik, N. F. Ndebele

**Affiliations:** 1 Portsmouth Hospitals University NHS Trust; 2 A2i, Hampshire and Isle of Wight Healthcare NHS Foundation Trust, Portsmouth, United Kingdom

## Abstract

**Introduction:**

The presence of a bidirectional relationship between low folate and depression has long been demonstrated. Folate deficiency has been shown to be associated with worse depressive symptoms, worse outcomes of treatment, and increased chance of relapse. Conversely, patients with depression have been shown to be more likely to have a lower than normal folate. The idea of providing folate supplementation as an adjuvant therapy for depression has been suggested, even discounting the physical health benefits of folate replacement in deficiency. In September 2015, it was published that 14.7% of adults in the UK have a serum folate lower than ~4.5ug/L (Buttriss, J.L. *Nutrition Bulletin* 2015; 40(3),153–157.), while staff at our service noted anecdotally that prevalence seemed to be higher in our patient population.

**Objectives:**

To assess the proportion of the patients within a secondary care adult mental health service that have a folate deficiency, compare this to other measures of diet to review whether this is as a result of dietary deficiencies, and to assess whether there is an association between a diagnosis of depression, and a decreased folate level, within our patient population.

**Methods:**

Data was manually collected via the electronic records of patients included (all patients aged 18-65 seen in the service physical health clinic between 20/09/24 and 25/04/25 who had blood haematinics taken and a valid folate level result. Their blood tests were recorded, including folate, B12, sodium, potassium, and ferritin). Their letters and records were searched to collect the mental health diagnoses of each patient.

**Results:**

In our patient population, we found that 37% of our patients had a folate deficiency, and a further 10% had a borderline deficiency (defined as <3.0μg/L and <4.0μg/L respectively, n=90). Mean serum folate was 5.91μg/L. There was no association found between folate deficiency and deficiencies of sodium, potassium, or vitamin B12. Pearson’s Rank Correlation Coefficient between folate levels and ferritin levels returned a p value of -0.05. Mean folate in patients with depression was 5.55μg/L against 6.23μg/L for patients without depression. Folate levels in patients with and without a depression diagnosis returned a point biserial correlation one-tailed p value of 0.31.

**Conclusions:**

Despite a lack of data on folate deficiency prevalence and average folate levels in the UK and globally, as well as inconsistent use of methods or units to measure folate levels in existing data, these results suggest a higher prevalence of folate deficiency within our patient population. There is no suggestion from this data that this higher prevalence is due to overall malnutrition. Finally, there is a weak to moderate correlation between depression and lower serum folate levels, consistent with the observed difference in group means, and in line with some of the previous literature.

**Disclosure of Interest:**

None Declared

